# Objective Monitoring of Tablet Use–Related Optical Exposure and Its Association With Axial Length in Preschool Children: Cross-Sectional Intelligent Monitoring Study

**DOI:** 10.2196/79266

**Published:** 2026-01-28

**Authors:** Yidong Zhu, Hao Chen, Senlin Lin, Hong Jiang, Mingdao Zhang, Yi Sun, Chenshu Li, Yingnan Jia

**Affiliations:** 1 Preventive Medicine and Health Education Department, School of Public Health, Fudan University, 130 Dong An Road, Shanghai, 200032, China, 86 13764665540; 2 Shanghai Eye Diseases Prevention & Treatment Center, Shanghai, China; 3 Shanghai Eye Hospital, School of Medicine, Tongji University, Shanghai, China; 4 Department of Maternal, Child and Adolescent Health, School of Public Health, Fudan University, Shanghai, China; 5 Midea Group (China), Nanchang, Jiangxi, China

**Keywords:** screen brightness, ambient illuminance, axial length, preschool children, visual health

## Abstract

**Background:**

In recent years, the global prevalence of myopia among children has continued to rise. The preschool years represent a critical period for visual development, and the widespread adoption of electronic screens among young children has brought increasing attention to pediatric visual health. However, the association between visual environmental exposures related to screen use—such as screen brightness and ambient illuminance—and the risk of myopia in preschool children has not been thoroughly investigated.

**Objective:**

This monitoring study aimed to investigate the association between electronic screen brightness, ambient illuminance, and axial length in preschool children to provide evidence-based support for developing screen brightness usage recommendations for this population.

**Methods:**

This cross-sectional monitoring study was conducted between March and July 2023 in Shanghai, China, involving 2 representative samples of kindergarten children aged 3 to 6 years. Each participant was provided with a tablet preinstalled with intelligent monitoring software, which continuously and objectively recorded real-time data on screen time and screen brightness over a consecutive 7-day period. In addition, comprehensive data collection encompassed standardized ophthalmic assessments, high-precision ambient illuminance measurements, simulated laboratory lighting evaluations, and parental questionnaires. Associations between ambient illuminance, screen brightness, and axial length were analyzed using multivariable linear regression and restricted cubic spline models.

**Results:**

Of the 199 children included in the total sample, 124 (62.3%) were boys, and 75 (37.7%) were girls. After adjustment for demographic characteristics, parental myopia, and screen use behaviors, the median ambient illuminance during tablet use was significantly inversely associated with axial length (β=–0.13, 95% CI –0.22 to –0.04; *P*=.006). A nonlinear dose-response relationship was identified between median screen brightness and axial length (*P*_nonlinearity_=.004), with axial elongation accelerating beyond approximately 27 cd/m² and peaking around 56 cd/m². Boys (*P*<.001) and greater height (*P*=.33) were also significantly associated with longer axial length.

**Conclusions:**

Higher ambient illuminance during tablet use is associated with shorter axial length in preschoolers, whereas screen brightness exhibits a nonlinear effect on axial elongation. This study highlights the importance of optimizing both environmental lighting and device settings to protect visual health in young children, providing empirical support for guidelines on safe digital device use and ambient lighting conditions in early childhood.

## Introduction

The rising global prevalence of myopia among children and adolescents has become a significant public health challenge [1]. Early intervention is critical, as a younger age of onset is strongly associated with a higher risk of developing high myopia [2,3]. Axial length represents a key biomarker for evaluating myopia severity and predicting disease progression, with abnormal elongation being strongly associated with myopia advancement [4]. The preschool stage (age 3‐5 y) is a critical period for refractive development. During this time, the characteristics of visual environmental exposure can profoundly influence the onset and progression of myopia.

In our digitalized society, electronic screens have become a dominant component of children’s visual environment. A large-scale epidemiological study conducted across 38 countries on 6 continents revealed that 60% to 93% of children and adolescents are exposed to screens for more than 2 hours daily [5], with preschool children showing a trend of earlier initial screen exposure and higher usage frequency [6,7]. This underscores the urgent need to evaluate the potential impact of screen exposure on visual health and myopia risk in preschoolers. Population-based studies have preliminarily demonstrated the association between screen time and myopia. A systematic review and meta-analysis published in *The Lancet Digital Health* confirmed that the independent duration of smartphone and tablet use as well as the cumulative effect of screen time from computers were significantly associated with an increased risk of myopia [8]. A study from Japan further identified a relationship between prolonged screen time and abnormal axial length elongation in young children [9]. A cross-sectional study from the Netherlands revealed a significant positive correlation between the increased frequency of uninterrupted smartphone usage lasting over 20 minutes and increased scleral elasticity ratio as well as the anterior chamber depth-to-lens thickness ratio [10]. Although existing studies have investigated the association between screen time and visual development, research on the impact of screen brightness, a critical optical parameter, on visual health remains absent and warrants further exploration.

Modern mobile devices are commonly equipped with ambient light sensors that enable automatic screen brightness adjustment, a dynamic feature that may influence the visual system through two mechanisms: first, the adaptation of screen brightness to ambient light levels may affect visual function; and second, excessively high ambient light levels may induce dry eye symptoms, while abnormally elevated screen brightness could increase the burden of glare perception [11-13]. Evidence from animal models indicates that low illumination levels can induce myopia, whereas increased light exposure may activate retinal dopaminergic activity (primarily via the D2 receptor pathway), thereby regulating retinal gene expression and suppressing the biological signaling pathways that promote axial elongation [14]. Furthermore, ergonomic research has identified an inverted U-shaped relationship between display brightness and visual performance, fatigue, and discomfort, suggesting that both excessively low and high brightness levels compromise visual comfort and performance [15]. Benedetto et al [11] demonstrated that increased screen brightness significantly exacerbates visual fatigue. However, epidemiological evidence regarding the impact of screen brightness on eye axial elongation in children remains lacking.

Therefore, the primary aim of this study was to collect real-time objective exposure data through intelligent monitoring technology and then examine the association between electronic screen brightness, ambient illuminance, and axial length in preschool children. These findings may contribute to the development of evidence-based guidelines regarding screen use and myopia prevention in young children.

## Methods

### Study Design and Population

This cross-sectional monitoring study was conducted in two districts of Shanghai, China: Xuhui District (urban) and Pudong New Area (suburban), from March to July 2023. A random sampling method was used to select a kindergarten from each of the 2 districts. For each kindergarten, a cluster sampling method was used to select 5 classes, in which the average number of children in each class was 30, from the junior, middle, and senior grades. A total of 199 preschool children participated in the study, each of whom was provided with a tablet for a 1-week period. Before participation, parental consent was obtained during the eligibility screening phase. During the monitoring period, participants were instructed to maintain their typical screen usage habits, with the provided tablets being strictly limited to use by child participants only, thereby ensuring the integrity of exposure data collection. The study enrollment criteria were established as follows. Inclusion criteria were as follows: (1) preschool children currently registered in the kindergarten; (2) aged 3 to 6 years at the time of recruitment; and (3) whose parents or other caregivers have allowed them to use screen devices, such as televisions, mobile phones, tablets, and computers. Exclusion criteria were as follows: (1) presence of diagnosed organic ocular pathologies, (2) demonstrated inability to complete refractive error assessments, and (3) absence of legally authorized guardian consent.

### Measures

#### Vision Screening

Axial length, corneal curvature, and refractive errors were measured using standardized ophthalmic instruments. Spherical and cylindrical refractive errors were assessed with the KR-800 auto-refractor (Topcon Corporation), while axial length and corneal curvature were measured using the IOL Master 700 (Carl Zeiss Meditec AG). To ensure measurement accuracy, examinations were conducted sequentially, starting with the right eye, followed by the left eye. If children demonstrated poor cooperation, measurements were repeated. All examiners received prior training, and instruments were regularly calibrated to maintain precision.

#### Screen Exposure Monitoring Technology

The *Healthy Screen Viewing for Children (HSVC)* application, developed in collaboration with Huawei Corporation, was installed on dedicated tablets provided to participants [16]. This software automatically recorded screen usage data, including application type, screen brightness, and duration of use, at 1-minute intervals over a 7-day monitoring period. Equipped with built-in ambient light sensors, the tablets dynamically adjusted screen brightness according to real-time ambient lighting conditions. The HSVC application concurrently recorded the corresponding screen luminance values (in cd/m²) along with precise time stamps for each measurement. All collected data were securely transmitted to a cloud server for subsequent analysis.

#### Simulated Lighting Environment Setup

To convert the recorded screen brightness values into corresponding ambient illuminance levels for each child, we established a response model between screen brightness and horizontal illuminance under controlled laboratory conditions. This model allowed us to estimate the ambient illuminance during each child’s tablet use based on the screen brightness data captured by the HSVC application. A controlled experiment was conducted in a professional lighting laboratory equipped with blackout conditions, where a response model between horizontal illuminance and screen brightness was constructed. The lighting environment was simulated using a warm-white light source with a correlated color temperature of 3000 K and a color rendering index (Ra) >80, to reflect typical nontask lighting conditions found in residential settings. The relative spectral power distribution of the light source is provided in Multimedia Appendix 1. This setup was designed to ensure both environmental realism and comfort.

The self-luminous device was operated under its default automatic brightness adjustment mode and fixed on a standard platform, with the screen center positioned at a height of 75 cm above the ground. A CL-500A spectroradiometer (KONICA MINOLTA) was used to record illuminance levels, with the sensor placed horizontally at the center of the desktop, approximately 30 cm in front of the screen. In accordance with the BS ISO/CIE 8995-1:2025 standard, horizontal illuminance was adopted as the primary photometric parameter due to its experimental stability and representativeness in evaluating screen-based visual environments. A total of 32 paired measurements of screen brightness and horizontal illuminance were collected by systematically adjusting ambient light intensity. A significant nonlinear relationship was observed between screen brightness and horizontal illuminance. As illustrated in Multimedia Appendix 2, the fitted quadratic regression model was as follows:


$$\begin{array}{c} E=0.199x^{2}-11.862x+177.930 \end{array}$$

where x denotes the screen brightness (unit: cd/m²) and E represents the corresponding horizontal illuminance (unit: lux). The model exhibited a high goodness of fit (*R*^2^=0.988).

On the basis of the illuminance requirements specified in BS ISO/CIE 8995-1:2025 for *computer work only and young children* (300 lux) and *general classroom activities* (500 lux), the experimental data were classified into three ambient lighting categories: low illuminance (<300 lux), representing relaxed or recreational settings; moderate illuminance (300‐500 lux), suitable for light visual tasks or display-based activities; and high illuminance (>500 lux), corresponding to functionally enhanced or brightly lit environments.

#### Covariates

A self-administered online questionnaire was distributed to parents to collect demographic information, including the child’s age, sex, caregiver characteristics, only-child status, parental education levels, and household income. Additionally, the questionnaire collected relevant covariates based on existing literature, such as parental myopia status and children’s daily behaviors (eg, duration of indoor and outdoor activities and sedentary time). Furthermore, the questionnaire assessed screen time across all media types, addressing the limitations of the HSVC in capturing data from television, personal computers, and other nontablet devices.

### Statistical Analysis

Categorical variables were analyzed through frequency distributions (%), whereas continuous variables were presented according to their distribution. The normality of all continuous variables was assessed using the Shapiro-Wilk test. The Shapiro-Wilk test indicated that axial length and height were normally distributed (*P*>.05), whereas the other continuous variables deviated significantly from normality. Normally distributed data were presented as mean (SD), and nonnormally distributed data were presented as median (25th percentile and 75th percentile). Univariate analyses used independent *t* tests for binary comparisons (eg, sex differences) and 1-way ANOVA for multigroup comparisons (eg, age category). To evaluate monotonic relationships between variables, the Spearman rank correlation coefficient (ρ) was used. Spearman correlation coefficients quantified bivariate associations between axial length and continuous physiological or behavioral measures (eg, height, weight, and activity durations). Before model fitting, key regression assumptions were verified. The homoscedasticity assumption was evaluated using a Breusch-Pagan test, and the normality of residuals was formally assessed using the Shapiro-Wilk test; no substantial violations were detected (*P*>.05). Multivariable linear regression models adjusted for covariates (eg, age, sex, parental myopia, screen time, and activity levels) to assess independent effects of ambient illuminance and tablet brightness on axial length. Restricted cubic spline (RCS) models with 3 knots explored nonlinear dose-response relationships. Statistical significance was set at *P*<.05 (2 tailed), and analyses were performed using R software (version 4.3.2; R Foundation for Statistical Computing).

### Ethical Considerations

Before the participants underwent screen exposure monitoring and questionnaire surveys, we informed their parents (legal guardians) of the entire process of participation in the surveys as well as the benefits and risks involved, and the legal guardians signed the informed consent. As a participation acknowledgment, children received art supply kits valued at US $15 per set. This study conformed to the ethical guidelines of the 1975 Declaration of Helsinki and was approved by the Ethics Committee for Medical Research at the School of Public Health, Fudan University (IRB#2023-11-1088). When extracting data from the Huawei Research Platform and the online questionnaire library for analysis, all data were deidentified, and an anonymous study ID was used as an identifier for each participant.

## Results

### Participant Characteristics

Complete demographic and axial length data were available for all 199 participants. As presented in Table 1, 62.3% (n=124) of the participants were boys, and the majority (n=144, 72.4%) were aged between 4 years 9 months and 5 years 6 months. The participants’ caregivers were predominantly their mothers (n=106, 53.3%), and more than half of the parents held a bachelor’s degree (fathers: n=113, 56.8%; and mothers: n=113, 56.8%). The prevalence of maternal myopia was significantly higher (n=113, 56.8%) compared to paternal myopia (n=108, 54.3%), and maternal myopia was found to be significantly associated with a longer axial length (*P*=.01).

**Table 1.** Demographic characteristics and comparison of axial length between groups (N=199).

Variable	Values, n (%)	Axial length (mm), mean (SD)			*T* test/*F* test^a^ (*df*)	*P* value^b^
Sex					6.184 (197)	<.001
Boy	124 (62.3)	22.68 (0.653)				
Girl	75 (37.7)	22.07 (0.717)				
Age					3.163 (2)	.04
4 y 9 mo to 5 y 6 mo	144 (72.4)	22.52 (0.747)				
4 y 3 mo to 4 y 9 mo	38 (19.0)	22.32 (0.682)				
3 y 3 mo to 4 y 3 mo	17 (8.5)	22.11 (0.692)				
Caregiver					1.254 (3)	.29
Mother only	106 (53.3)	22.52 (0.789)				
Father only	11 (5.5)	22.58 (0.749)				
Grandparents	78 (39.2)	22.33 (0.662)				
Other family members	4 (2.0)	22.67 (0.664)				
Education of father					1.571 (2)	.21
Secondary school or less	22 (11.0)	22.30 (0.755)				
Senior high school	64 (32.2)	22.36 (0.768)				
Bachelor’s	113 (56.8)	22.53 (0.715)				
Education of mother					1.065 (2)	.35
Secondary school or less	32 (16.1)	22.27 (0.742)				
Senior high school	54 (27.1)	22.48 (0.812)				
Bachelor’s	113 (56.8)	22.48 (0.700)				
Total annual household income (CNY)					–1.503 (197)	.13
<200,000 (US $27,800)	84 (42.2)	22.36 (0.719)				
≥200,000 (US $27,800)	115 (57.8)	22.52 (0.749)				
Only-child status					–0.389 (197)	.70
Yes	101 (50.8)	22.43 (0.714)				
No	98 (49.2)	22.47 (0.766)				
Current marital status of parents					–1.697 (197)	.09
Married	192 (96.5)	22.43 (0.734)				
Single/long-term separation	7 (3.5)	22.91 (0.767)				
Whether the father is nearsighted					–0.498 (197)	.62
No	91 (45.7)	22.42 (0.735)				
Yes	108 (54.3)	22.47 (0.744)				
Whether the mother is nearsighted					–2.470 (197)	.01
No	86 (43.2)	22.30 (0.750)				
Yes	113 (56.8)	22.56 (0.713)				
Whether the child was ill in the past year					–1.167 (197)	.24
Yes	173 (86.9)	22.42 (0.740)				
No	26 (13.1)	22.61 (0.721)				

a *T* tests were used for binary comparisons, and the *F* statistic from a 1-way ANOVA was used for multigroup comparisons.

b *P* values were derived from independent *t* tests (for binary variables such as sex) or 1-way ANOVA (for multicategory variables such as age group). *P*<.05 was considered statistically significant.

Univariate analyses revealed significant differences in axial length based on sex, with boys having a greater mean axial length (22.68, SD 0.653 mm) compared to girls (22.07, SD 0.717 mm; *P*<.001). Additionally, significant differences in axial length were observed across different age groups (*P*=.04). However, no significant associations were found between axial length and variables, such as caregiver type, parental education level, household income, or only-child status (*P*>.05).

### Correlation Analyses

**Figure 1.**
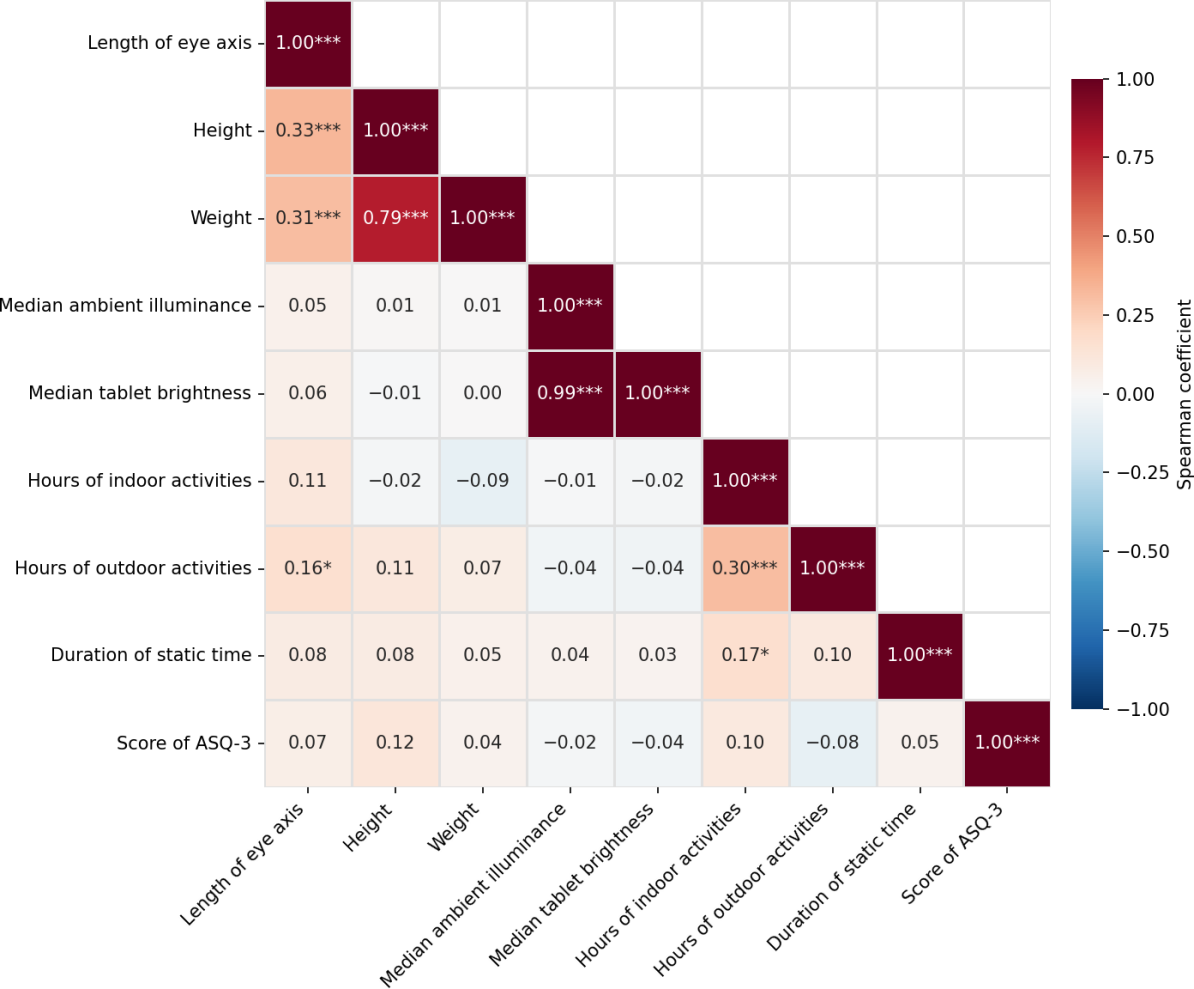
Spearman rank correlation heatmap of key variables and axial length (N=199). Note: *P<.05, **P<.01, ***P<.001. ASQ-3: Ages and Stages Questionnaires, Third Edition.

The Spearman rank correlation analysis revealed several significant associations between certain demographic and behavioral factors and axial length. Specifically, height (ρ=0.33; *P*<.001), weight (ρ=0.31; *P*<.001), and outdoor activity duration (ρ=0.16; *P*=.03), all demonstrated statistically significant positive correlations with axial length (Figure 1). Notably, these relationships persisted even after adjusting for potential confounders, including parental myopia status and digital device exposure parameters.

### Associations Between Ambient Illuminance or Tablet Brightness and Axial Length

As presented in Tables 2 and 3, multivariable linear regression demonstrated that higher median ambient illuminance during tablet use was inversely associated with axial length (β=–0.13, 95% CI –0.22 to –0.04; *P*=.006), after adjusting for sex, age, height, parental myopia, total screen time, and behavioral factors (Table 2). Specifically, the median ambient illuminance was 134.77 lux (P25=67.74 lux and P75=221.65 lux). Boy sex (*P*<.001) and greater height (*P*=.33) also significantly predicted axial length. However, there was no significant association between tablet brightness and axial length (Table 3). Although the median tablet brightness was 55 cd/m^2^ (P25=47 cd/m^2^ and P75=63.25 cd/m^2^), it did not exhibit a linear association with axial length (Table 3).

**Table 2.** Associations between ambient illuminance and axial length (N=199)^a^.

Variables	β^b^ (95% CI)		*P* value
Height (cm)	0.16 (0.01 to 0.31)		.03
Weight (kg)	0.05 (–0.08 to 0.18)		.46
Sex			
Boy	Reference		—^c^
Girl	–0.59 (–0.79 to –0.40)		<.001
Age			
4 y 9 mo to 5 y 6 mo	Reference		—
4 y 3 mo to 4 y 9 mo	–0.01 (–0.28 to 0.25)		.92
3 y 3 mo to 4 y 3 mo	–0.12 (–0.51 to 0.26)		.52
Whether the mother is nearsighted			
No	Reference		—
Yes	0.12 (–0.07 to 0.31)		.20
Whether the father is nearsighted			
No	Reference		—
Yes	0.09 (–0.09 to 0.28)		.33
Total screen time used (ks)	0.03 (–0.06 to 0.12)		.56
Median ambient illuminance (lux)	–0.13 (–0.22 to –0.04)		.006
Hours of indoor activities (min)	0.05 (–0.05 to 0.14)		.31
Hours of outdoor activities (min)	0.05 (–0.04 to 0.14)		.26
Duration of static time (min)	–0.01 (–0.10 to 0.08)		.85
Score of ASQ-3^d^ (pts)	0.07 (–0.02 to 0.16)		.13

a Results are derived from multivariable linear regression models. The dependent variable is axial length (mm).

b β represents the regression coefficient.

c Not applicable.

d ASQ-3: Ages & Stages Questionnaires, Third Edition.

**Table 3.** Associations between tablet brightness and axial length (N=199)^a^.

Variables	β^b^ (95% CI)		*P* value
Height (cm)	0.17 (0.02 to 0.32)		.02
Weight (kg)	0.04 (–0.09 to 0.17)		.51
Sex			
Boy	Reference		—
Girl	–0.58 (–0.78 to –0.39)		<.001
Age			
4 y 9 mo to 5 y 6 mo	Reference		—
4 y 3 mo to 4 y 9 mo	–0.01 (–0.28 to 0.26)		.94
3 y 3 mo to 4 y 3 mo	–0.10 (–0.49 to 0.29)		.60
Whether the mother is nearsighted			
No	Reference		—
Yes	0.12 (–0.07 to 0.31)		.22
Whether the father is nearsighted			
No	Reference		—
Yes	0.10 (–0.09 to 0.29)		.32
Total screen time used (ks)	0.03 (–0.06 to 0.12)		.51
Median tablet brightness (cd/m²)	–0.08 (–0.17 to 0.01)		.09
Hours of indoor activities (min)	0.05 (–0.05 to 0.14)		.35
Hours of outdoor activities (min)	0.06 (–0.04 to 0.15)		.24
Duration of static time (min)	–0.02 (–0.11 to 0.07)		.67
Score of ASQ-3^c^ (pts)	0.07 (–0.03 to 0.16)		.17

a Results are derived from multivariable linear regression models. The dependent variable is axial length (mm).

b β represents the regression coefficient.

c ASQ-3: Ages and Stages Questionnaires, Third Edition.

### Dose-Response Relationships Between Ambient Illuminance or Tablet Brightness and Axial Length

**Figure 2.**
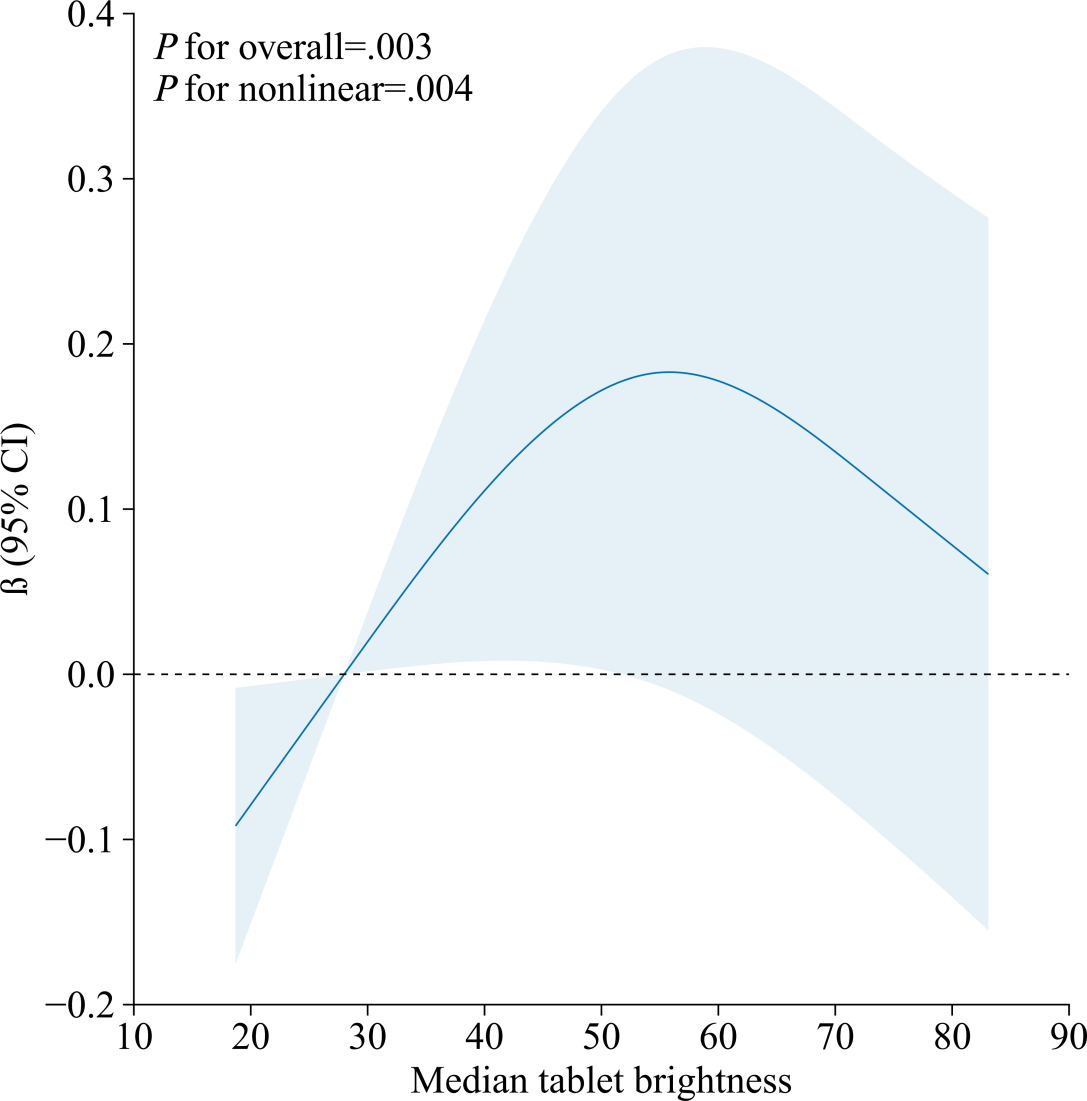
Dose-response relationship between tablet brightness and axial length (N=199). The solid line represents the estimated adjusted regression coefficient β, and the blue area indicates the 95% CI. Knots were placed at the 10th, 50th, and 90th percentiles of the median tablet brightness.

**Figure 3.**
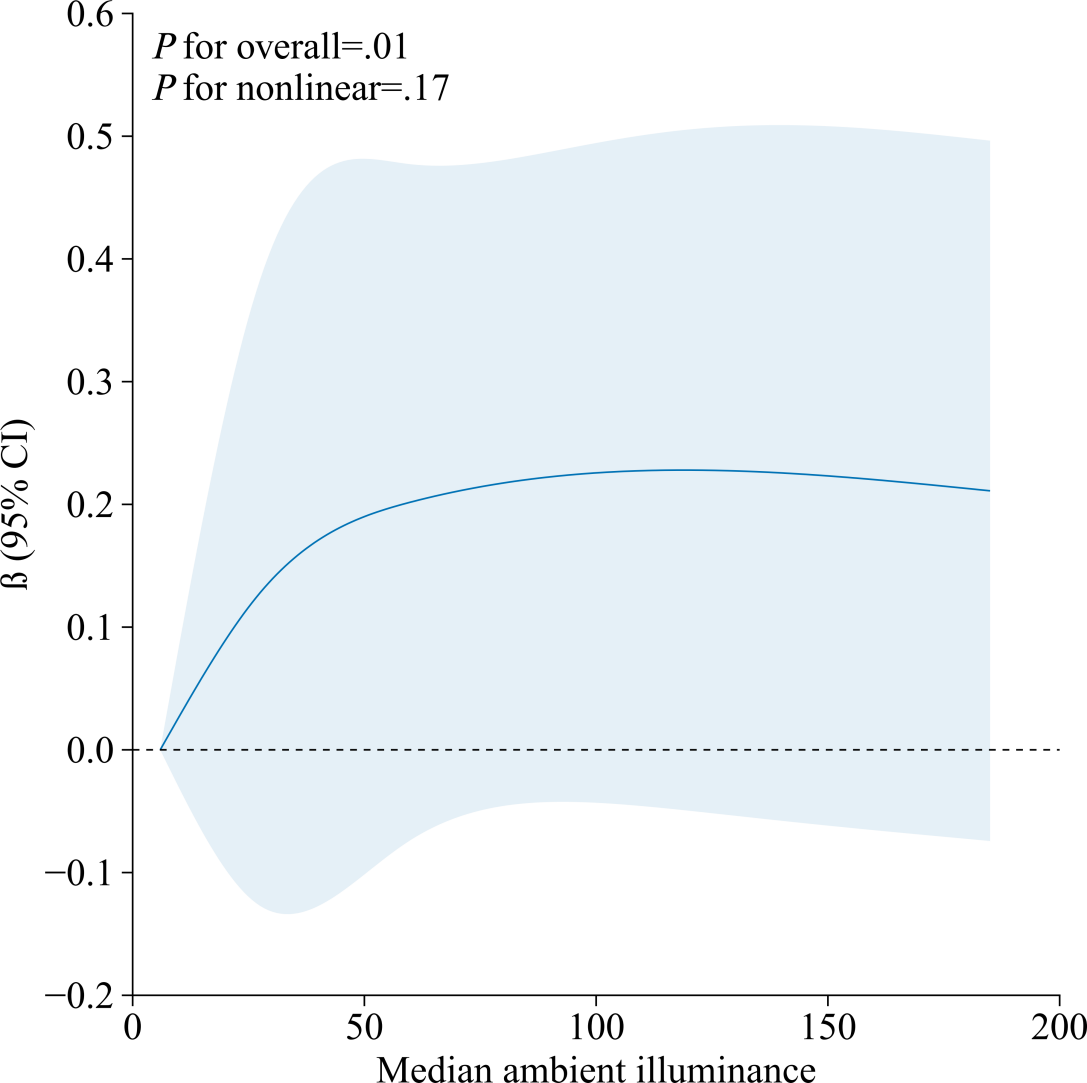
Dose-response relationship between ambient illuminance and axial length (N=199). The solid line represents the estimated adjusted regression coefficient β, and the blue area indicates the 95% CI. Knots were placed at the 10th, 50th, and 90th percentiles of the median ambient illuminance.

The dose-response relationships of tablet brightness and ambient illuminance with axial length were examined using RCS, as shown in Figures 2 and 3. Multivariable linear regression models incorporating RCS were fitted with 3 knots placed at the 10th, 50th, and 90th percentiles of the exposure distribution [17]. All models were adjusted for sociodemographic factors (eg, boy or girl, age, height, weight, and parental myopia), behavioral factors (indoor and outdoor activity duration, and sedentary time), and developmental characteristics (score from Ages and Stages Questionnaires, Third Edition). A significant nonlinear association was identified between median tablet brightness and axial length (*P*_nonlinearity_=.004). The coefficient β represents the marginal change in axial length (in mm) per unit increase in exposure. The fitted curve indicated a negative slope at brightness levels below 27 cd/m², suggesting a reduction in the rate of axial elongation. This was followed by a transitional phase (27‐56 cd/m²) where the curve became positive and increased, peaking at approximately 56 cd/m². Beyond this point, the fitted curve gradually decreased. In contrast, no significant nonlinear relationship was found between ambient illuminance and axial length (*P*_nonlinearity_=.17), with the curve remaining approximately flat across the measured range (Figure 3).

## Discussion

### Principal Findings

On the basis of continuous 7-day real-time monitoring data collected via the HSVC application from 199 preschool children aged 3 to 6 years in 2 kindergartens in Shanghai, this study investigated the associations among screen luminance, ambient illuminance during device use, and axial length in early childhood, addressing a critical gap in the existing literature regarding the impact of dynamic screen optical parameters on visual development in children. Our findings reveal a significant inverse association between higher ambient illuminance during tablet use and shorter axial length, as well as a nonlinear dose-response relationship between tablet luminance and axial length. These results elucidate the link between digital device optical exposure and axial elongation, providing robust empirical evidence to inform public health recommendations on screen use practices and ambient lighting standards in preschool children.

### Relationship Between Ambient Illuminance and Axial Length

Our study identified a significant inverse association between ambient illuminance during tablet use and axial length in preschool children, aligning with findings from animal models [14]. Experimental studies in chicks and rhesus monkeys have established that light intensity modulates myopic progression [18-21]. Multiple investigations further demonstrate that increased light exposure may exert its effect by activating retinal dopaminergic activity—primarily through D2 receptor pathways—thereby regulating retinal gene expression and suppressing pro-axial elongation signaling [22-25]. The retinal dopamine system is thus considered a potent inhibitor of ocular growth [26].

The present results align with several domestic studies investigating the role of light environment in myopia prevention. For instance, a study on elevated classroom lighting found that increasing illuminance significantly slowed axial elongation and reduced the incidence of myopia among school-aged children [27]. Similarly, a cluster randomized trial in Shanghai using objective monitoring showed that increased outdoor time and sustained high-intensity light exposure delayed axial elongation [28]. Together, these studies support a protective role of light exposure against axial growth. Our study extends this evidence from “macroenvironmental” settings to “microenvironments during screen use,” refining the understanding of light exposure effects.

Complementing this mechanistic context, it is important to recognize that not all studies of indoor optical environments have detected an effect on refractive development. For instance, a school-based comparison of high-daylight versus low-daylight classrooms reported no overall difference in 6-month changes in refractive error or axial length, with effects limited only to a baseline subgroup characterized by short axial length [29]. Similarly, an independent population-based analysis found no association between exposure to ambient nighttime lighting during infancy and later myopia [30]. These inconsistent outcomes may be attributable to methodological and contextual differences. The former study used classroom-level daylight factors—a distal and nonspecific metric—which may not accurately capture retinal light exposure during near-device use. Furthermore, the study was conducted in a specific ethnic and educational setting (Korean schoolchildren), where genetic predispositions and academic pressures may modulate the effects of light exposure. The latter study examined nighttime lighting exposure in infancy. This differs fundamentally from daytime ambient light during visual tasks in its spectral composition, exposure timing, and biological mechanisms. Furthermore, the cohort primarily comprised White participants, who have a distinct genetic background from Asian populations. These methodological and demographic differences may collectively account for the heterogeneity observed regarding the association between light exposure and myopia.

It is noteworthy that typical indoor illuminance experienced by humans rarely exceeds 1000 lux and is usually much lower, often within the range of 100 to 500 lux [31]. According to standards set by the International Organization for Standardization and the International Commission on Illumination, as well as China’s national guidelines (GB 7793‐2010) on classroom lighting, the recommended threshold for children’s indoor ambient illuminance is 300 lux [32,33]. In our findings, the median ambient illuminance during tablet use for 174 children was below 300 lux, with an overall median of 134.77 (IQR 67.74-221.65) lux, indicating that the ambient lighting conditions when participants used electronic devices were generally lower than internationally recommended standards. Nevertheless, we still observed a robust association between ambient illuminance and axial length, underscoring the critical role of environmental lighting in ocular development. Suboptimal lighting may fail to provide adequate visual comfort and could contribute to visual fatigue or other adverse effects on visual development. The widespread prevalence of insufficient ambient lighting during device use is concerning, as it represents a potentially modifiable environmental risk factor for myopia in children. Given the increasing reliance on digital devices in early childhood, our findings highlight the urgent need for evidence-based guidelines on ambient lighting for children to mitigate potential risks to visual health and development.

However, our results diverge from some previous reports, suggesting that ambient illuminance has no significant effect on visual fatigue [34]. This discrepancy suggests that the mechanisms underlying short-term visual discomfort and long-term regulation of axial growth may differ and warrant separate investigation. While short-term visual fatigue is likely associated with transient visual stimuli and ocular muscle strain, long-term axial elongation is influenced by a complex interplay of factors, including light-mediated regulation of ocular signaling pathways. From the perspective of long-term axial development, our study provides novel evidence for the impact of ambient illuminance on children’s visual health.

### Relationship Between Tablet Brightness and Axial Length

Using RCS with knots at the 10th, 50th, and 90th percentiles of the exposure distribution, we identified a significant nonlinear association between median tablet brightness and axial length after adjustment for sociodemographic, behavioral, and developmental covariates (*P*_nonlinearity_=.004). At lower levels of tablet brightness (with a median tablet brightness <27 cd/m²), increases in brightness are accompanied by a deceleration in axial length growth (β<0), suggesting that very dim screens may accelerate eye growth, whereas moderate brightness increases could help mitigate excessively rapid axial elongation. When tablet brightness exceeds a certain threshold (median tablet brightness >27 cd/m²), further increases in brightness result in an acceleration of axial elongation, with excessively high screen brightness stimulating abnormal axial growth. When tablet brightness reaches a critical threshold (median tablet brightness=56 cd/m²), the rate of axial elongation peaks. Excessive brightness can cause glare or strong light stimuli, disrupting normal physiological eye development and accelerating axial elongation, which may worsen myopia progression [13]. In this study, the median tablet brightness was 56 cd/m² (P25=47 cd/m² and P75=63.25 cd/m²), falling within the critical range that may influence ocular growth.

These findings align with the “parabolic or inverted U-shaped dose-response curve” described in previous ergonomic studies regarding screen brightness, visual performance, and discomfort symptoms [15]. At very low brightness, children may reduce viewing distance to maintain visibility, increasing accommodative demand and potentially stimulating axial elongation. Moderately increased brightness may improve legibility and allow for more comfortable viewing distances, corresponding to the observed negative marginal effect below 27 cd/m² [35,36]. Within the midrange, heightened brightness during sustained near-work may provide stronger visual signals associated with axial elongation, resulting in a positive marginal effect that peaks near 56 cd/m² [35,37]. At even higher brightness, discomfort and incipient glare may prompt self-regulatory behaviors—such as microbreaks, blinking, or adjustment of posture or gaze—that could attenuate the marginal effect, consistent with the decline in the fitted curve at high brightness levels [37-39]. This nonmonotonic pattern aligns with ergonomic literature in which display brightness often exhibits nonlinear relationships with both performance and discomfort [15,40]. To our knowledge, this is the first study to quantitatively characterize a nonlinear dose-response relationship between objectively measured tablet brightness and axial length in children, addressing a significant evidence gap in this field. This finding is also consistent with the “light dose-axial plasticity” effect reported in domestic studies on optical interventions. For instance, a randomized clinical trial in 10 primary schools in Shanghai demonstrated that repeated low-level red light therapy reduced the annual axial elongation rate in children from 0.47 mm to 0.30 mm over 12 months, alongside a significant decrease in new-onset myopia [41]. Although repeated low-level red light represents a therapeutic intervention fundamentally distinct from daily screen-based illuminance, its outcomes clearly indicate that the mode and dosage of light stimulation can structurally influence axial elongation. This lends biological plausibility to the nonlinear association between screen brightness and axial length observed under naturalistic conditions in our study and further supports the notion that optical parameters play a modifiable and nonlinear role in axial eye growth.

More importantly, the study identifies a relatively “safe” brightness range for children’s use of mobile devices, within which axial elongation remains stable and the impact on myopia progression is minimal. To translate these key findings into practical application, device manufacturers should implement child-specific brightness-limiting features that cap maximum screen luminance below 56 cd/m², incorporating ambient-adaptive safeguards to prevent exceedance of this threshold and thus reduce the risk of excessive axial elongation. Caregivers should be advised to maintain screen brightness within the empirically derived safe range identified in this study and to encourage regular, timed breaks during prolonged use to minimize potential adverse effects on visual development. These results provide robust scientific evidence to support public health guidelines for optimal screen brightness settings in both educational and household environments.

### Limitations

Several limitations of this study should be acknowledged. First, the cross-sectional nature of this study precludes any causal inferences. Future prospective studies are needed to determine whether reduced ambient illuminance directly accelerates axial elongation and to clarify causal relationships among relevant factors. Second, our sample was limited to children in Shanghai, potentially restricting the generalizability of our findings to populations with different screen usage habits. Future research should extend to larger and more diverse cohorts across various regions and populations to enhance external validity. Additionally, although we attempted to control for covariates, such as the duration of outdoor activities and unmeasured confounders—including genetic predisposition and dietary factors—may partially account for the observed associations. Future research should address these factors to comprehensively elucidate the determinants affecting axial eye development in children. Finally, as cycloplegic refraction was not performed in this study, the accuracy of refractive error measurements was limited. Therefore, axial length was selected as the primary outcome. In future research, we will refine the methodology by incorporating cycloplegic refraction to enable a more comprehensive evaluation of the effects of ambient illuminance on children’s visual health.

### Conclusions

This monitoring study demonstrates a significant association between both ambient illuminance and tablet screen brightness with ocular axial length in preschool children, with a nonlinear dose-response relationship observed for screen brightness. These findings underscore the importance of optimizing both environmental lighting and device settings to safeguard early childhood visual development and inform evidence-based guidelines for digital device use in preschool children.

## Supplementary material

10.2196/79266 Multimedia Appendix 1 Relative spectral power distribution of the light source (correlated color temperature=3000 K).

10.2196/79266 Multimedia Appendix 2 Nonlinear response relationship between screen luminance and horizontal illuminance of the self-luminous device.

## Abbreviations

HSVC: Healthy Screen Viewing for Children
RCS: restricted cubic spline
