# Supplementary material for: Objective Monitoring of Tablet Use–Related Optical Exposure and Its Association With Axial Length in Preschool Children: Cross-Sectional Intelligent Monitoring Study
Source: JMIR Hum Factors. 2026 Jan 28;13:e79266. doi: 10.2196/79266 (PMC12851525; doi:10.2196/79266)
Supplement: Multimedia Appendix 2 [file humanfactors-v13-e79266-s002.docx]

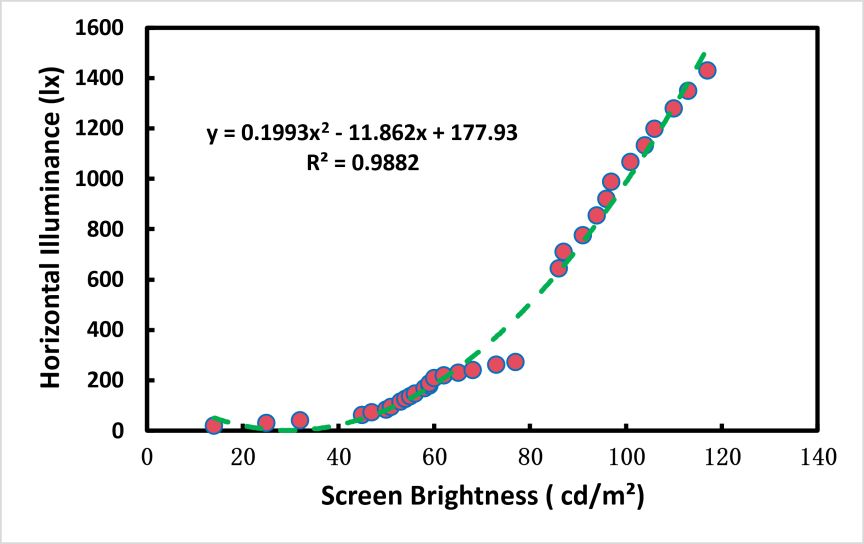


***Figure S2. Nonlinear response relationship between screen luminance and horizontal illuminance of the self-luminous device.***
